# Characteristics of split-step skills of the world’s top athletes in badminton

**DOI:** 10.1371/journal.pone.0316632

**Published:** 2025-01-03

**Authors:** Hidehiko Shishido, Takeshi Nishijima

**Affiliations:** 1 Soka University, Hachioji, Japan; 2 Tokyo Metropolitan University, Hachioji, Japan; Shahid Chamran University of Ahvaz, ISLAMIC REPUBLIC OF IRAN

## Abstract

**Objective:**

The purpose of this study was to quantitatively measure the split-step skills of the world’s top badminton players to clarify the characteristics underlying these skills when moving into the forehand position in the rear court.

**Methods:**

We analyzed the four best ranking players (1^st^ to 4^th^) in the men’s singles competition at the World Badminton Federation (BWF) World Championships 2023, a world tournament whose match videos are available online. Analysis 1 was conducted to determine the location of the players’ feet on the court when performing a split-step while moving to the forehand rear court, as well as the width of the stance and the reaction time from that stance to taking the first step. To define the characteristics of top athletes, the split-step skill performance of these athletes was evaluated during play. Analysis 2 was used to determine whether the performance of the split-step when moving to the forehand rear court varied depending on the position of the opposing player.

**Results:**

Analysis 1 showed that the split-step position was gathered close to the base, with an average split-step reaction time of 0.25 s and a split-step stance width comprising 50% of the players’ height. These results were similar among all top players evaluated. Analysis 2 showed that the difference in the number of shuttlecocks that hit the opponent’s backhand rear court (LR) affected their degree of split-step skill.

**Conclusion:**

In this study, we quantitatively measured the split-step skills of the world’s top badminton athletes and clarified the characteristics of their positioning into the forehand rear court during active play. Herein, movement and performance analysis using match videos available online was used to gain novel insights into the performance of these athletes.

## 1. Introduction

Determining performance indices for professional athletes using footage of active play in competition can provide insights from a perspective different from that obtained through analysis in a laboratory environment [1]. In recent years, recordings of international matches have become widely available on online platforms, such as YouTube. As a result, research has begun to be conducted using these videos in their analysis [2]. Match videos published on online platforms have a high resolution, enabling high-precision motion analyses. In badminton competitions, research targeting international matches published on online platforms has been conducted and useful information has been obtained [3]. However, few examples of such cases exist at present.

In badminton, a player loses a point if the shuttlecock lands on court. Even if a player has the skill to return it, failure to reach the shuttlecock in time results in a point for the opponent. Players must quickly anticipate where the shuttlecock will land and position themselves to return it effectively. This requires precise footwork. Players can minimize unnecessary movements and save energy by mastering appropriate footwork techniques. Foot movement is a skill among badminton players [4]. Footwork mainly consists of side stepping and cross stepping [5]. In this context, the faster the step speed and reaction time, the more advantageous the footwork movement [6, 7]. Recent studies have examined the effects of footwork training on agility [8] and reaction time [9], and effective footwork training programs have been proposed to improve performance [10, 11]. The split-step constitutes an initial form of footwork in badminton, consisting of a small step taken immediately before the opponent hits the shuttlecock and serves to smooth the transition to a rapid footwork action by placing the body’s center of gravity in a neutral position [12, 13]. Thus, the split-step plays an important role in the initial movement of footwork and has been a research target from various perspectives; however, the split-step technique of top athletes has yet to be comprehensively analyzed.

Top athletes, regardless of sport, perform at an extraordinarily high level, and their techniques and strategies often serve as models for others. Careful observation and analysis of athletes’ movements and forms during competitions allows coaches to identify specific tactics and areas for improvement and tailor the most effective coaching methods for each individual. Research on elite athletes is not only vital for enhancing performance in competitive sports but also for advancing scientific knowledge, providing valuable insights that can be applied across various fields. For example, in badminton, techniques and strategies are closely linked and improving both can significantly elevate a player’s performance.

Studies on top athletes have already provided important insights, such as race analysis of a male marathon runner in two different races and brain activity analysis of a male soccer player [1, 14]. Therefore, similar studies on top badminton players should be equally valuable.

In this study, the split-step skills of the world’s top badminton athletes were quantitatively measured to elucidate the characteristics of this skill. This included clarifying where the feet were on the court when performing the split-step, how wide the stance was, and how long the reaction time was from the stance to the first step during a match. A measurement technique was applied comprised of manually pointing out the position of a player’s thenar and converting this into the actual size of the overhead image of the court using match videos published on an online platform. This method could be applied to all badminton game videos available online as long as the video was shot from a fixed camera from a bird’s eye viewpoint. In this study, the position of interest was the players in movement to the forehand rear court. This position is useful for analysis because it is often the first technique that beginners learn.

## 2. Methods

### 2.1. Match to be analyzed

In this study, recordings of the world badminton championships were used. The four best ranked players (1^st^ to 4^th^) in men’s singles of the World Badminton Federation (BWF) World Championships 2023 were included in our analysis. Match videos were obtained from videos officially released by the BWF [15]. Therefore, the following seven matches were included in the analysis:

1. **Final:** Kunlavut Vitidsarn vs. Kodai Naraoka
2. **Semi-final:** H.S. Prannoy vs. Kunlavut Vitidsarn, Kodai Naraoka vs. Anders Antonsen
3. **Quarter finals:** Anders Antonsen vs. Kenta Nishimoto, Kunlavut Vitidsarn vs. Wang Tzu Wei, Shi Yu Qi vs. Kodai Naraoka, and Viktor Axelsen vs. H.S. Prannoy

A summary of the results [16] of the men’s single matches and the world rankings of the corresponding players [17] is provided in Table 1.

**Table 1:** Profiles of target players.

Country	Player	BWF World Ranking (2023/12/26)	BWF World Championships 2023	Height[cm]	Dominant arm
**THA**	**Kunlavut VITIDSARN(VITI)**	**7**	**GOLD**	**177**	**Right**
**JPN**	**Kodai NARAOKA(NARA)**	**2**	**SILVER**	**173**	**Right**
**DEN**	**Anders ANTONSEN(ANTO)**	**9**	**BRONZE**	**188**	**Right**
**IND**	**PRANNOY H. S. (PRAN)**	**8**	**BRONZE**	**179**	**Right**

The sample size was not determined in advance. The target athletes for this study were defined as those in the top 10 world rankings.

### 2.2. Extracting match scenes for analysis

To clarify the characteristics of the split-step skill, the point at the start of a match when the player moves to the forehand rear court was analyzed.

**Fig 1:**
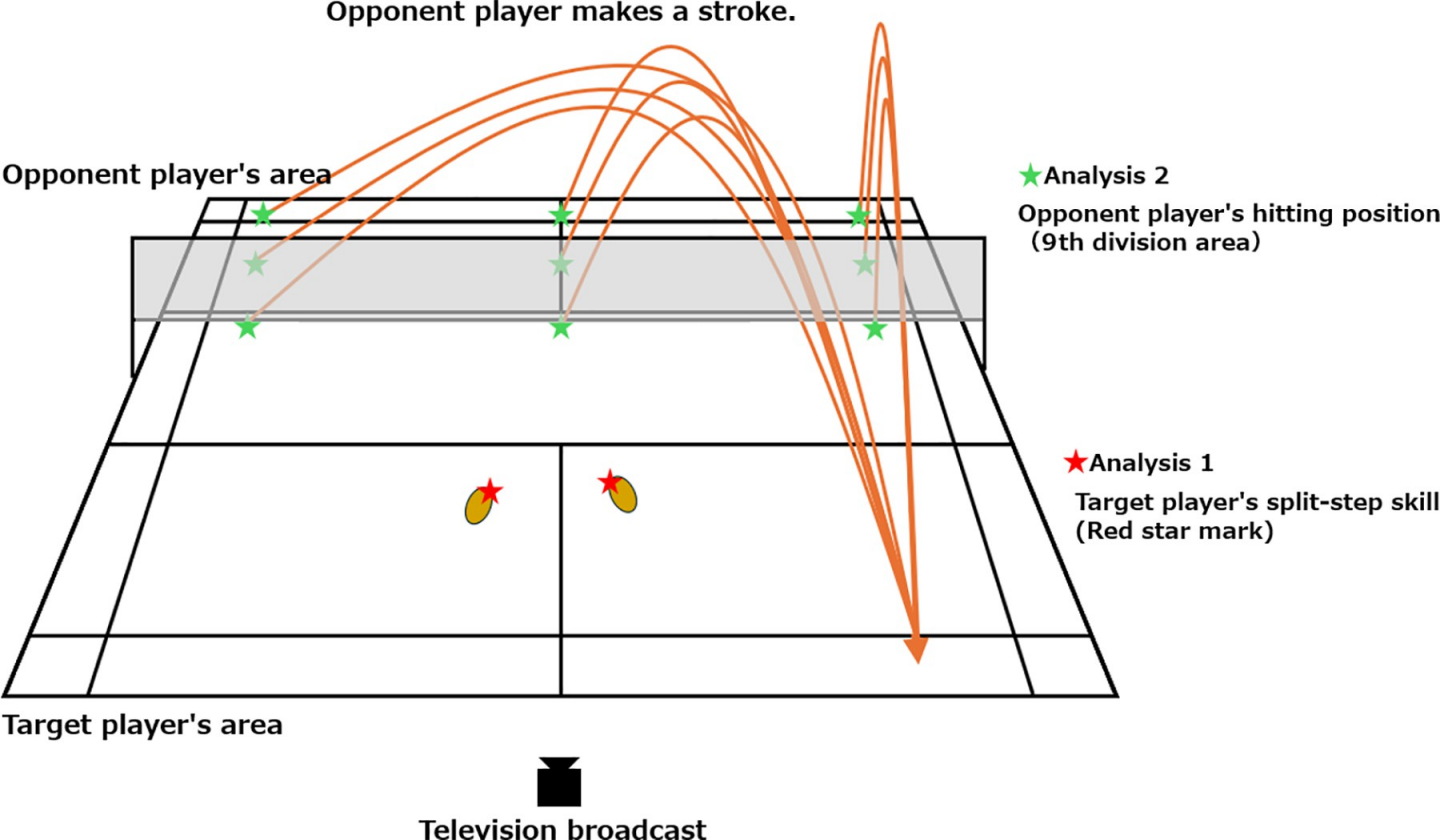
Summary of analysis 1 and 2.

A summary of the analyses is provide in Fig 1: (1) analysis 1 was used to determine the superiority or inferiority of top athletes’ split-step skill performance during a game by measuring the location of their feet on the court when performing the split-step moving into the forehand rear court, the width of the player’s stance, and the length of the reaction time from the stance to taking the first step; (2) analysis 2 was used to clarify whether the performance of the split-step skill depended on the hitting position of the opponent when moving to the forehand rear court.

In this study, the area of the court in front of the camera, which was less affected by coordinate transformation errors, was selected as the target area for extraction (the area at the back of the court was excluded from the analysis). Homography transformation, which can transform the image coordinates [px] to actual measurement values [m], was used for the analysis.

### 2.3. Measuring the feet position in the split-step stance

The positions of both feet in the split-step stance were measured at thenar of both feet in the split-step stance of the target player at the moment when the opponent player hit the shuttlecock to the forehand rear court (Fig 1, red asterisk). To this end, the match recordings were divided into 30 static frames per second (30 fps). The static frame in which the opponent hit the shuttlecock to the forehand rear court, as indicated by the asterisk in Fig 1, was selected. The image coordinates of the thenars of both feet of the target player were manually obtained (marked) for the static frame.

### 2.4. Measuring split-step stance width and midpoint (from pixels to meters)

**Fig 2:**
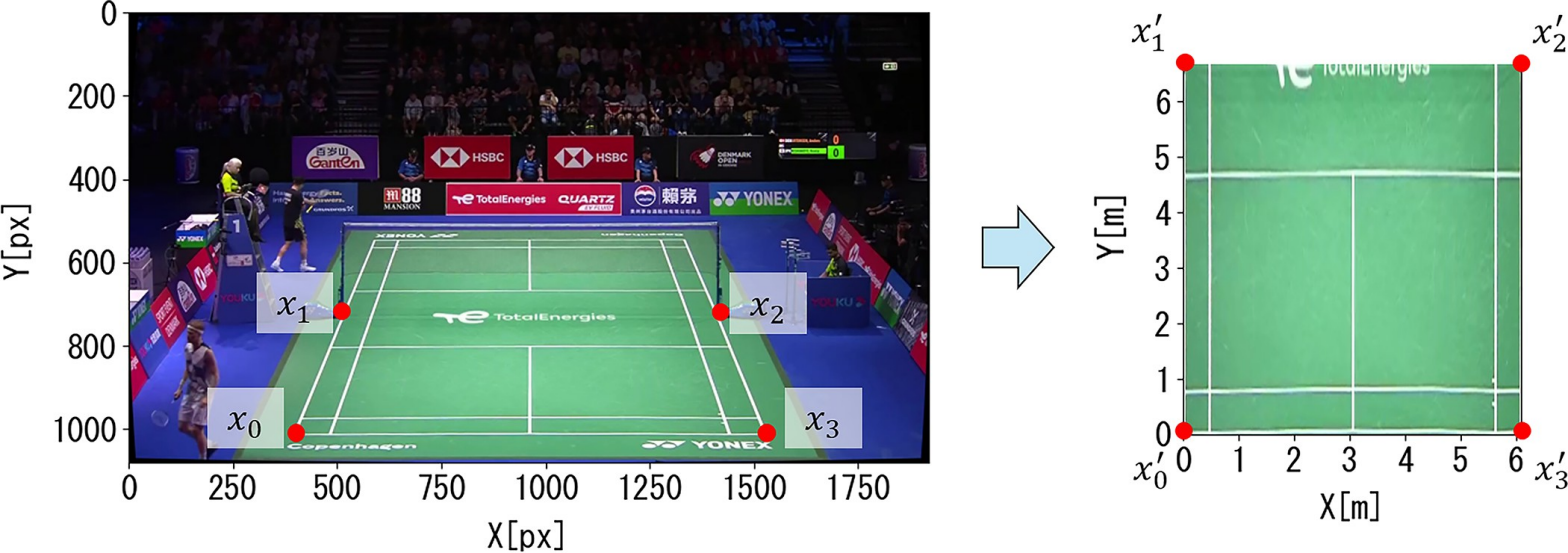
An overhead view image was generated by applying homography transformation to the badminton match recordings ([px]→[m]).

The measured coordinates of the left and right thenars were converted into an overhead view using a homographic transformation. For this method, homographic transformation, which converts image coordinates [px] into actual measurements [m], was utilized. Homographic transformation is an image processing technique that transforms an arbitrary quadrilateral image into another. As shown in Fig 2, applying a homography transformation to the badminton court image allows conversion to an overhead image of the badminton court. This homography transformation can be represented as follows: given the coordinates before transformation (*x*, *y*) and after transformation (*x*′,*y*′), with S as the scale parameter, it can be expressed as follows:

$$s(\begin{array}{c} x^{\prime }\\ y^{\prime }\\ 1 \end{array})=(\begin{array}{ccc} a & b & c\\ d & e & f\\ g & h & 1 \end{array})(\begin{array}{c} x\\ y\\ 1 \end{array})$$ (1)

Expanding Eq (1), it can be expressed as follows.


$$\{\begin{array}{c} x^{\prime }=\frac{ax+by+c}{gx+hy+1}\\ y^{\prime }=\frac{dx+ey+f}{gx+hy+1} \end{array}$$ (2)

In this way, the coordinates after homography transformation can be found.

Eq (2) was transformed to determine the unknowns (a–h) of the homography transformation matrix:

$$\{\begin{array}{c} x^{\prime }(gx+hy+1)=ax+by+c\\ y^{\prime }(gx+hy+1)=dx+ey+f \end{array}$$ (3)

$$\{\begin{array}{c} ax+by+c-gxx^{\prime }-hyx^{\prime }=x^{\prime }\\ dx+ey+f-gxy^{\prime }-hyy^{\prime }=y^{\prime } \end{array}$$ (4)

Substituting the coordinates before transformation with the four points after transformation:

$$\{\begin{array}{c} ax_{0}+by_{0}+c-gx_{0}x_{0}^{\prime }-hy_{0}x_{0}^{\prime }=x_{0}^{\prime }\\ dx_{0}+ey_{0}+f-gx_{0}y_{0}^{\prime }-hy_{0}y_{0}^{\prime }=y_{0}^{\prime }\\ ax_{1}+by_{1}+c-gx_{1}x_{1}^{\prime }-hy_{1}x_{1}^{\prime }=x_{1}^{\prime }\\ dx_{1}+ey_{1}+f-gx_{1}y_{1}^{\prime }-hy_{1}y_{1}^{\prime }=y_{1}^{\prime }\\ ax_{2}+by_{2}+c-gx_{2}x_{2}^{\prime }-hy_{2}x_{2}^{\prime }=x_{2}^{\prime }\\ dx_{2}+ey_{2}+f-gx_{2}y_{2}^{\prime }-hy_{2}y_{2}^{\prime }=y_{2}^{\prime }\\ ax_{3}+by_{3}+c-gx_{3}x_{3}^{\prime }-hy_{3}x_{3}^{\prime }=x_{3}^{\prime }\\ dx_{3}+ey_{3}+f-gx_{3}y_{3}^{\prime }-hy_{3}y_{3}^{\prime }=y_{3}^{\prime } \end{array}$$ (5)

Expressing Eq (5) as a matrix:


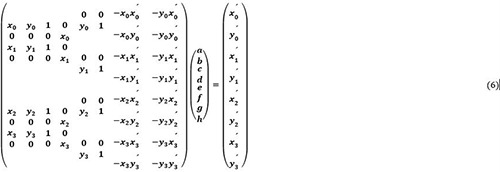


Transforming Eq (6) into an inverse matrix:


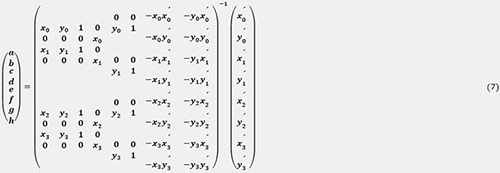


Unknowns (a–h) were obtained using Eq (7). In this case, the coordinates before transformation were obtained by taking four points on the court lines in the image coordinates. After the transformation, the coordinates were given by the four measured points specified in the badminton rules. In the homographic transformation shown in Fig 2, the position of the origin was denoted by $x_{1}^{\prime }$.

In this manner, by applying a homography transformation, the measured image coordinates of the left and right thenars were converted into an overhead view image, and the image coordinates [px] were converted into the actual measured values [m]. Therefore, the image coordinates of the thenar of both feet of the target player in the split-step stance could be converted into coordinates of the badminton court.

Next, the coordinates of the midpoints for the measured datasets of the left and right thenars after applying the homography transformation were calculated and aggregated. Then, the median for the aggregated midpoint datasets of the split-step stance was calculated. Finally, the distance between this median and the midpoint of the split-step stance was calculated, and the average and standard deviation of this distance are presented in Fig 2.

### 2.5. Accuracy verification of the measurement method for the split-step stance

**Fig 3:**
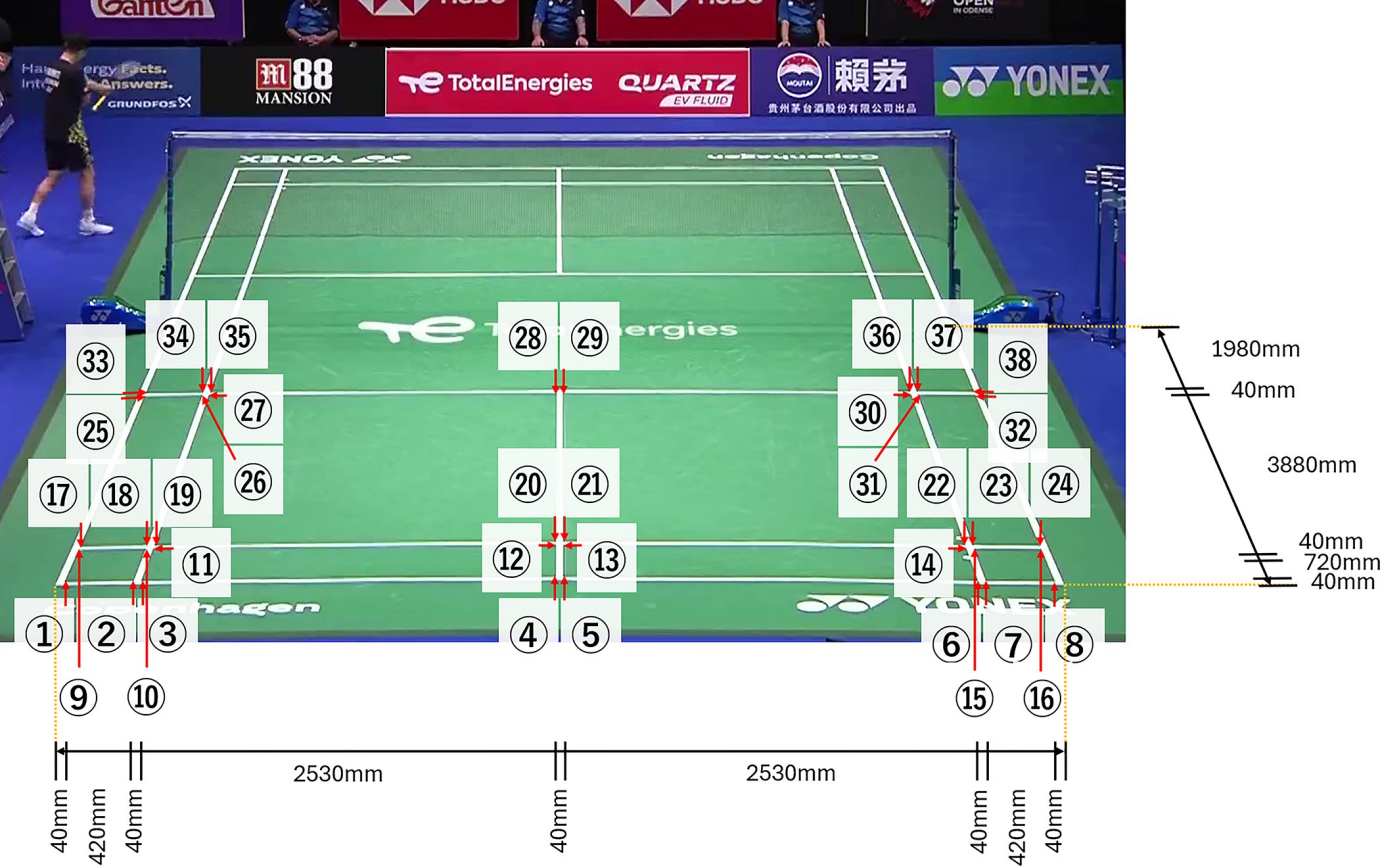
To verify the accuracy of the proposed method, 38 corner positions on the badminton court were used.

**Fig 4:**
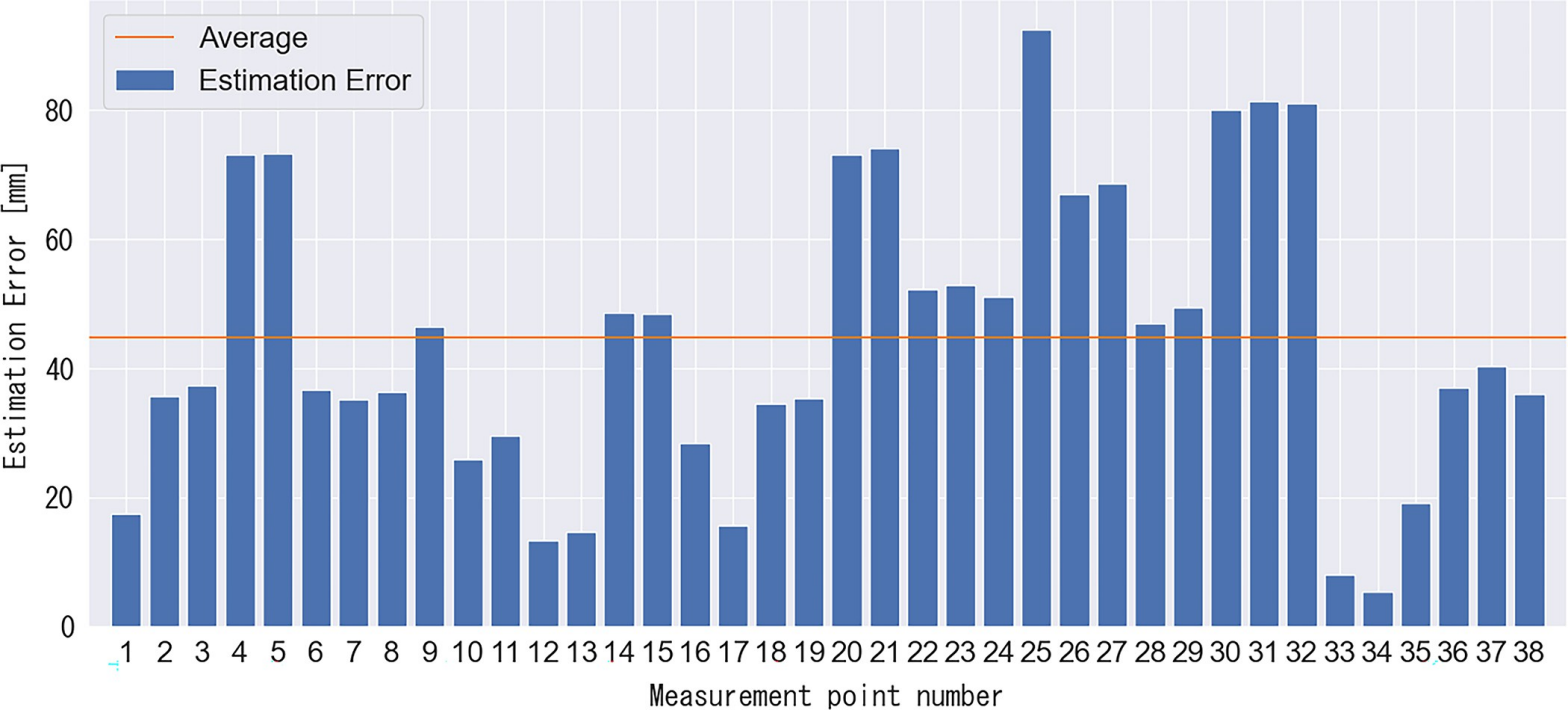
Comparison of the distances between the estimated 38 corner positions and the ground truth.

We conducted additional experiments to verify estimation accuracy, as outlined below. Estimation accuracy represents the error between the estimated values obtained using the proposed method and actual positions in the real world. In Section 2.4, we tested the accuracy of the proposed method for converting the image coordinates into an overhead view. In the validation experiments, we pre-acquired four points (Fig 2: x1, x2, x3, and x4) using the proposed method. We verified the estimation accuracy for the 38 corner positions, as shown in Fig 3. As regulations define the dimensions of a badminton court, we can calculate the true values for the 38 corner positions. Therefore, we compared the corner positions estimated using the proposed method with the true values. The comparison results are shown in Fig 4. The average estimation error of the proposed method was 44.8 ± 22.6 mm. Given the 40 mm width of the badminton court lines, it is evident that the conversion error of the proposed method falls within this range. Furthermore, the error ranged from a minimum of 5 mm to a maximum of 92 mm, suggesting that the proposed method for converting image coordinates into an overhead view is viable for data analysis of badminton footage.

Based on the above validation, we conclude that the data obtained using the proposed method has high reliability.

### 2.6. Measuring the reaction time

The reaction time of the target player was measured as the time from which the split-step was initiated at the moment the opponent player hit the shuttlecock to the forehand rear court, as shown in Fig 1 (red asterisk), until one foot of the target player left the ground. The target match recordings were divided into 30 static frames per second (30 fps). Next, the number of static frames from the start of the split-step stance until one foot left the ground was measured. The static frame interval was 0.03 seconds because the frames were split at 30 fps. Therefore, the reaction time was calculated by multiplying the number of static frames measured by 0.03.

### 2.7. Measuring the width of the split-step stance

The width of the split-step stance was calculated using the Euclidean distance between the two points from the left and right thenar coordinates. The split-step stance width/height ratio was calculated from the aggregated split-step stance width data using the height data of the athlete.

### 2.8. Measuring the number of shots

To determine whether the performance of the split-step skill differed depending on the opponent’s hitting position, we measured the opponent’s hitting position as shown in Fig 1 (green asterisk). In this case, the opponent’s shuttlecock trajectory was directed toward the forehand rear court. The number of shuttlecocks that hit the opponent’s area was recorded by observing the relevant scenes extracted from the match recordings. The court was divided into six sections: “front,” “mid,” and “rear,” in the direction of the depth of the sideline; “right,” “center,” and “left” in the direction horizontal to the net. The measured data were visualized using a heatmap.

### 2.9. Statistical analysis

#### 2.9.1, Analysis 1

The values of the mean and standard deviation (SD) were validated using one-way analysis of variance (ANOVA) using a significance level of 5%. If an effect was observed, multiple comparisons were performed using the Tukey-Kramer method. The data for statistical analyses are shown below.

- Euclidean distance data between the median and the midpoint of the split-step stance for the split-step stance data group
- Reaction time data
- Split-step stance width data

#### 2.9.2. Analysis 2

The total was calculated from the number of shots in each of the nine areas, and the proportions were obtained. The proportion of each area was verified using a difference test between the proportions in the two groups with a significance level of 5%. The data used for the statistical analysis are shown below:

- Number of hitting positions of the opponent player (nine split areas)

## 3. Results

The number of scenes analyzed is shown in Table 2. VITI had 43 scenes, NARA had 63 scenes, ANTO had 15 scenes, and PRAN had 42 scenes. ANTO was excluded from the analysis because of its low number of scenes compared with the other three players.

**Table 2:** Number of measurements for the target scene.

Country	Player	Scenes number of rear area of the forehand			
		final	Semi-final	Quarter finals	total
**THA**	**Kunlavut VITIDSARN(VITI)**	**12**	**21**	**10**	**43**
**JPN**	**Kodai NARAOKA(NARA)**	**42**	**12**	**9**	**63**
**DEN**	**Anders ANTONSEN(ANTO)**	**#**	**8**	**7**	**15**
**IND**	**PRANNOY H. S. (PRAN)**	**#**	**20**	**22**	**42**

### 3.1 Analysis 1: Results of reaction time analysis

**Fig 5:**
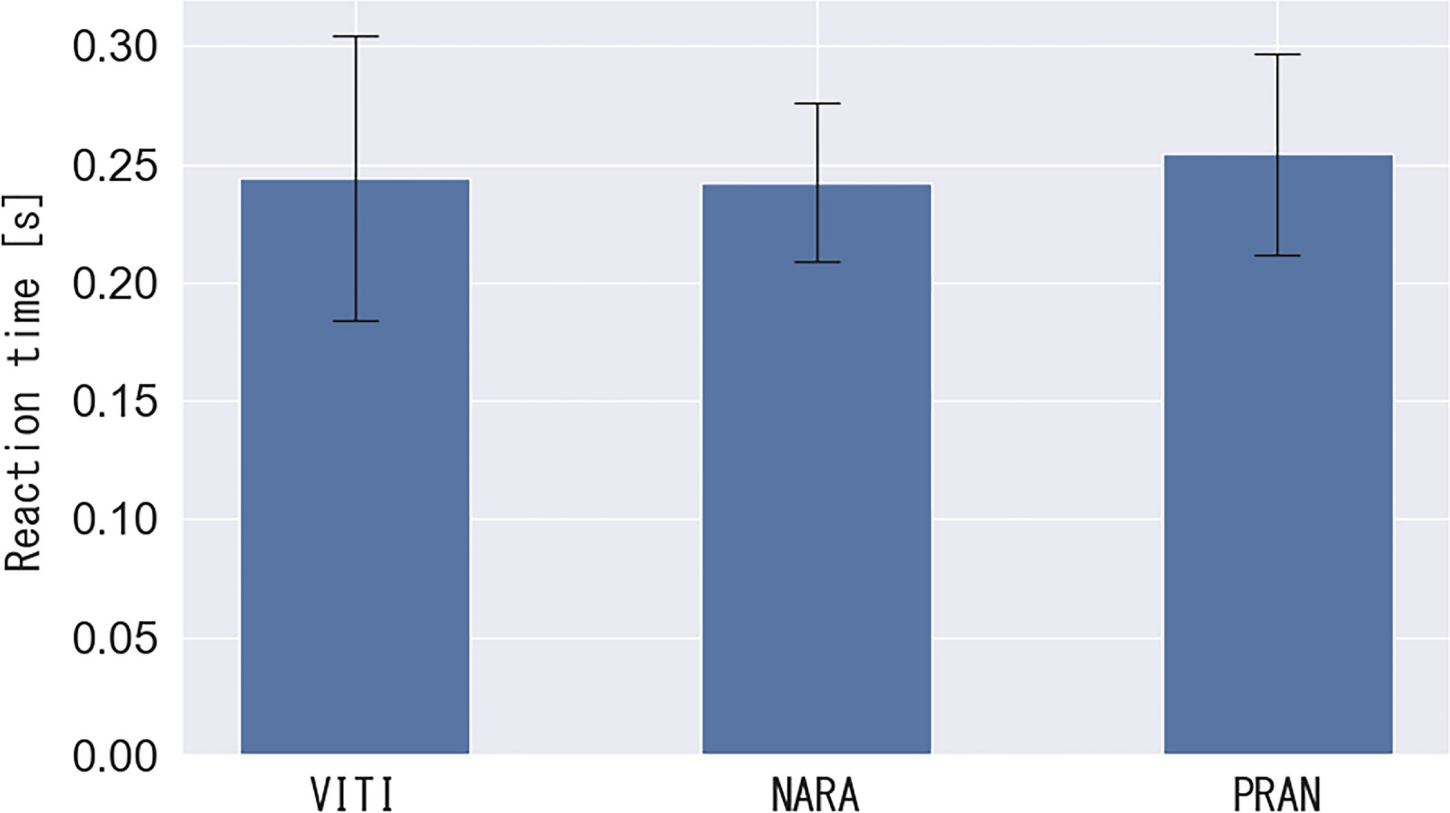
Reaction time to the rear area of the forehand.

The experimental results for the reaction times of the split-step are shown in Fig 5. The reaction time in VITI, NARA, and PRAN was 0.24 ± 0.06, 0.24 ± 0.03, and 0.25 ± 0.04 s, respectively. One-way ANOVA revealed no statistical differences between players (F (2, 145) = 3.06, p = 0.40).

### 3.2 Analysis 1: Results of split-step stance width analysis

**Fig 6:**
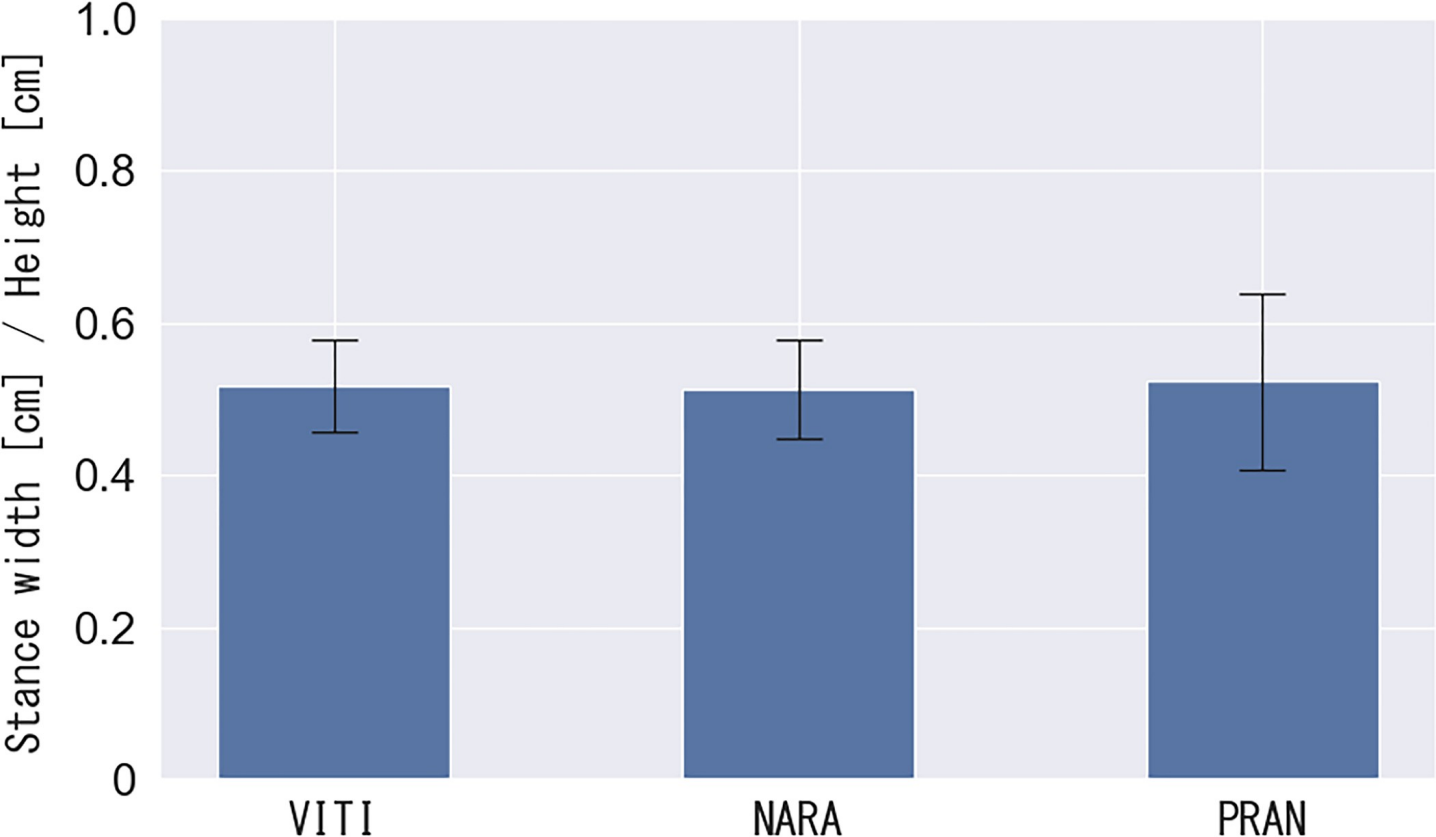
Stance width [cm]/height [cm].

The experimental results for the position of both feet (stance width) of the athlete in the split-step stance to the forehand rear court are shown in Fig 6. The split-step stance width in VITI, NARA, and PRAN was 0.52 ± 0.06, 0.51 ± 0.06, and 0.52 ± 0.11, respectively. One-way ANOVA revealed no statistical differences between the players (F (2, 145) = 3.06, p = 0.86).

### 3.3 Analysis 1: Results of split-step position analysis

**Fig 7:**
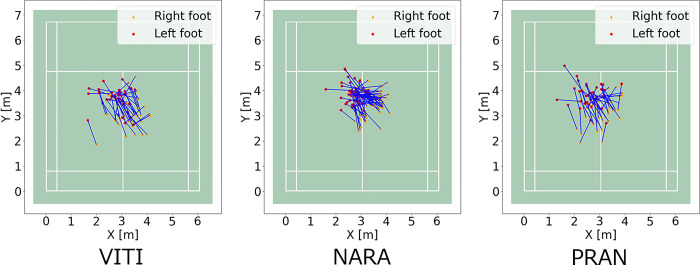
Split-step stance position to the rear area of the forehand (thenar).

**Fig 8:**
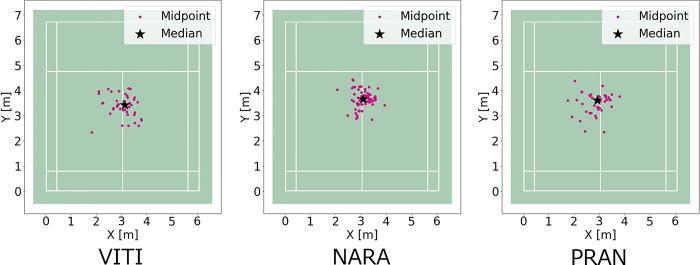
Aggregation of the midpoints of the split-step stance positions. The median is indicated by an asterisk.

**Fig 9:**
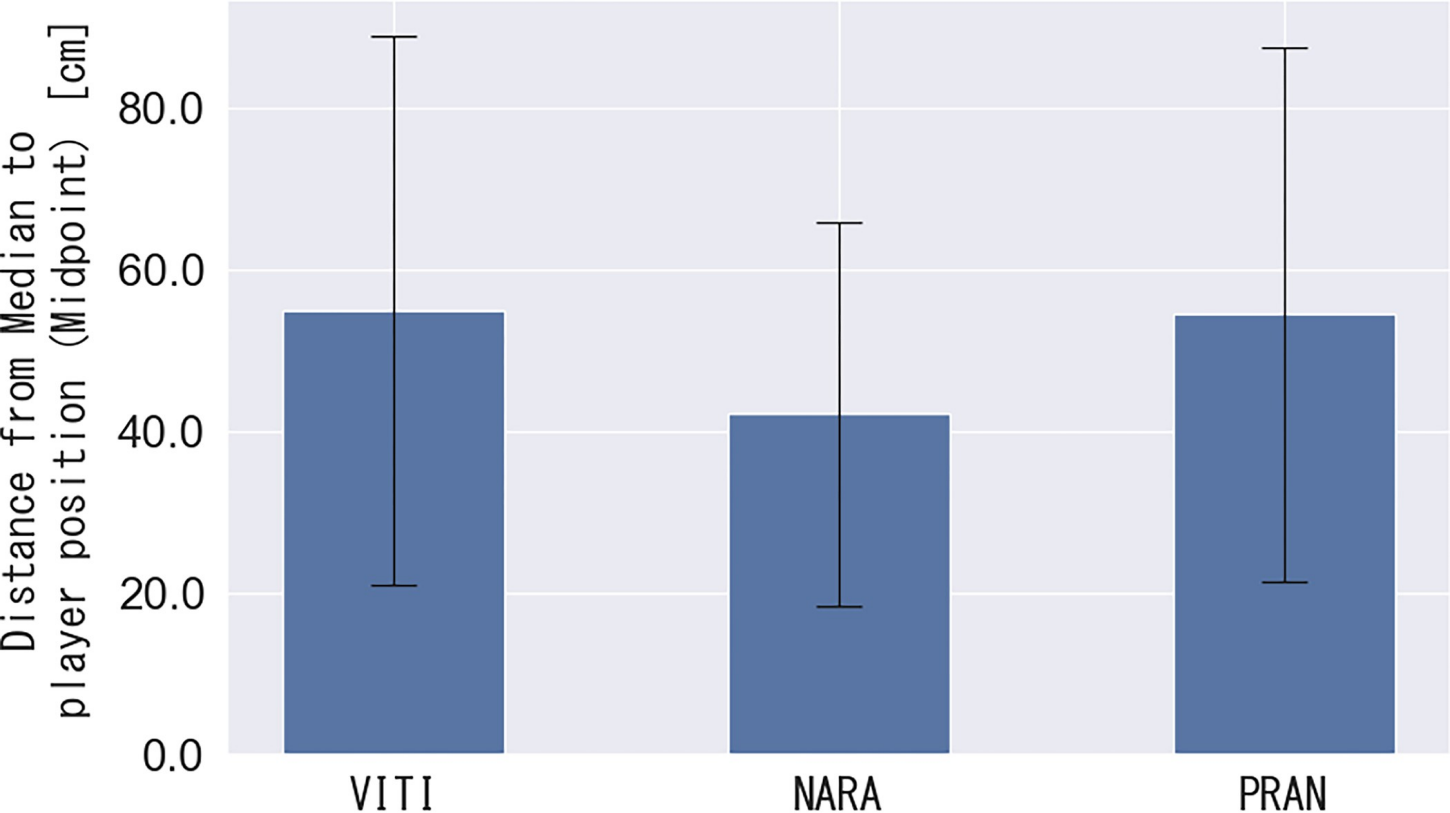
Distance from the median to the player’s position (midpoint).

The aggregated split-step stance positions of the left and right thenars with homographic transformation are shown in Fig 7. Fig 8 shows the results of aggregating the midpoints of the split-step stance positions. The median value of the midpoint of the split-step stance is plotted as an asterisk, denoting the distance between the median and the midpoint of the split-step stance position. As shown in Fig 9, the split-step position in VITI, NARA, and PRAN was 55.0 ± 34.3, 42.1 ± 23.9, and 54.5 ± 33.5 cm, respectively. One-way ANOVA revealed statistical differences between players (F (2, 145) = 3.06, p = 0.04). However, post-hoc multiple comparisons indicated no significant differences between any pair of players (p > 0.05).

### 3.4 Analysis 2: Effect of the opponent player’s hitting position on the split-step

**Fig 10:**
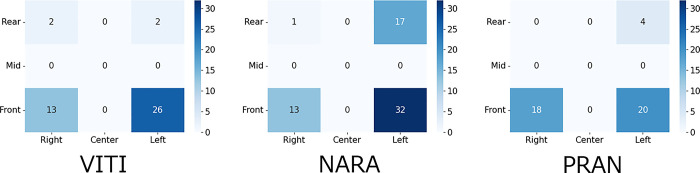
Number of measured hitting positions of the opponent player (nine segmented areas).

**Fig 11:**
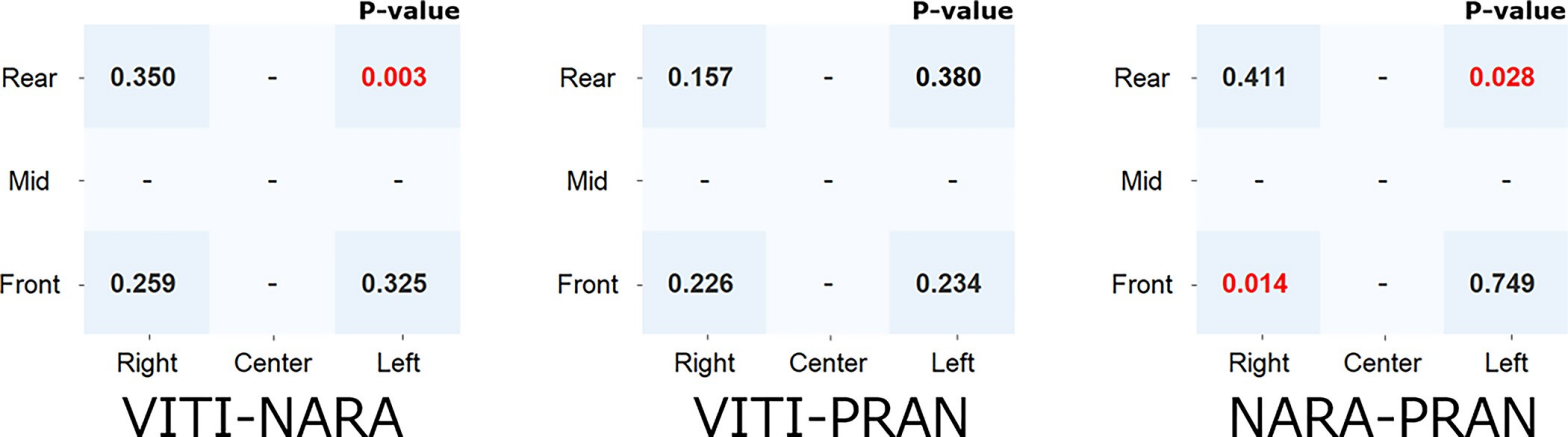
Differences in the ratios between the two groups for the proportion of each area of the opponent players hitting positions (nine segmented areas, P-value).

The number of opponent players hit positions measured is shown in Fig 10. An analysis of the difference in the proportion of hitting positions measured in each area between the two groups showed a significant difference in the LR area between the VITI and NARA players (P < 0.01). Additionally, significant differences were observed in the LR and RF areas between players with NARA and PRAN (p < 0.03). As shown in Fig 11, no significant differences were observed in other areas (p > 0.05).

## 4. Discussion

In this study, the split-step skills of the world’s top badminton athletes were quantitatively measured using recordings of their matches to clarify the characteristics of the split-step skills from the starting point during which athletes move to the forehand rear court. The superiority and inferiority of the performance of the split-step skills of all athletes were analyzed during a match by clarifying where the feet were on court when performing the split-step (i.e. where the feet were positioned on the court), the width of their stance, and the length of the reaction time from that initial stance to the first split-step. The superiority or inferiority of the top athletes’ performance in split-step skills during the game was analyzed.

Traditional performance and movement analyses have largely relied on data collected in laboratory settings, where researchers can carefully control the experimental conditions, allowing for a precise examination of how specific factors affect performance. However, this approach has several limitations. A significant drawback is that laboratory-based research cannot replicate the dynamic and unpredictable nature of real-life matches. In particular, laboratory simulations fail to capture the complexity of an actual game regarding real-time decision-making or player adaptations to changing conditions.

In contrast, analyzing real match footage reflects the true conditions of competition, including the unpredictable elements of gameplay. However, this method introduces numerous uncontrolled factors that researchers cannot control. Despite these challenges, the data gathered from real match footage provides more realistic and actionable insights into player and team performance in actual match settings. Thus, the use of match footage offers unique value that laboratory studies cannot fully replicate.

Although both controlled laboratory studies and real match footage analyses each have limitations, these approaches are complementary. This study recognizes the challenges inherent in analyzing real match footage, while highlighting valuable practical insights that can be derived from such data.

Analysis 1 examined the characteristics of the split step, specifically the position of the feet on the court, width of the stance, and reaction time from the stance to one foot leaving the ground. The split step was typically performed near the base and the stance width was 50% of the player’s height, a consistent pattern among the top three players. Additionally, the split step was performed with an average reaction time of 0.25 seconds, which was equivalent to that of 16-year-old male badminton players with experience (top-class junior players in Poland) [9]. As the level of competition increases, predicting and reading opponents’ shots become increasingly challenging. However, the ability to make quick decisions and initiate movements in such a brief period demonstrates world-class performance. This suggests the existence of an upper limit for the reaction time in badminton, which can be reached in the junior period. The valuable data on the reaction time and stance width obtained from Analysis 1 offer critical scientific insights, contributing to a deeper understanding of this field and potentially serving as a foundation for further research in related areas.

In the movement comprising the split-step stance position to the forehand rear court, one-way ANOVA revealed statistical differences between the players. However, post-hoc multiple comparisons indicated no significant differences between the pairs of players. A trend toward differences between NARA and the other two players was also observed.

Analysis 2, namely the analysis of the effect of the opponent’s hitting position on the split-step, was used to identify the contributing factors. As shown in Figs 10 and 11, there was a significant difference in the LR area between VITI and NARA and a significant difference in the LR and RF areas between NARA and PRAN. This suggests that NARA distributes more shots to the backhand rear (LR) than to the other two players. The fact that NARA hit more shots with longer trajectory times suggests that he used this time to return to the base and had ample time to prepare for the return. Therefore, we can consider that NARA’s split-step stance position was denser at the base than those of the other two players.

The results of Analysis 2 indicate that variations in shot placement are likely influenced by tactical factors such as managing fatigue, resetting the rally, or transitioning from a defensive to an offensive position. The relationship between shot placement and split-step execution underscores the critical role of combining techniques and strategies at the highest levels of competition. In badminton, in which techniques and tactics are deeply interconnected, these findings offer valuable insights for enhancing performance at the elite level.

Thus, by measuring the players’ positions (both feet) by using recordings of the matches available on an online platform, we were able to clarify the characteristics of the split-step skills of the world’s top players during the match, which had not been previously possible. The measurement method was a two-dimensional to three-dimensional (vertical direction 0) transformation, which proved useful for this analysis. This method could be applied to all videos of badminton matches published on online video platforms, as long as the videos are captured by a fixed camera from a bird’s eye perspective. Therefore, motion and performance analyses using publicly available game videos show potential in their contribution to improving competitive performance.

## 5. Conclusions

The results presented in this study highlight the potential of conducting movement and performance analyses that measure the positions of players (i.e. both feet) using recordings of matches that are widely available on online platforms. This type of analysis could provide valuable, novel insights into the performance of top athletes in different sports.

This study aimed to assess quantitatively the split-step technique of world-class athletes, focusing specifically on their characteristics during movements toward the forehand rear court. Although motor control and physiological factors are key components, this study extended beyond these aspects by analyzing publicly available match footage to gain new insights into athletes’ movements and performance. The core of this research is to examine the techniques and tactics players use to optimize their performance by drawing on data extracted from these match videos. Specifically, this study sought to understand how factors ranging from motor control and technical elements revealed through video analysis to tactical decisions interact with and influence a player’s overall performance. By adopting a comprehensive approach that goes beyond merely studying physiological or biomechanical mechanisms, this study focuses on a broader picture of athletic performance. This holistic perspective is a distinctive and innovative feature of this study, which offers new insights that traditional methods often overlook.

## Supporting information

S1 Dataset (XLSX)
